# Leadership Principles to Decrease Psychological Casualties in COVID-19 and Other Disasters of Uncertainty

**DOI:** 10.1017/dmp.2020.395

**Published:** 2020-10-22

**Authors:** George S. Everly, Albert W. Wu, Carolyn J. Cumpsty-Fowler, Deborah Dang, James B. Potash

**Affiliations:** Department of International Health, Johns Hopkins University Bloomberg School of Public Health, Baltimore, MD; Department of Psychiatry and Behavioral Sciences, Johns Hopkins University School of Medicine, Baltimore, MD; Department of Health Policy and Management, Johns Hopkins University Bloomberg School of Public Health, Baltimore, MD; Department of Medicine, Johns Hopkins University School of Medicine, Baltimore, MD; Johns Hopkins Hospital and Health System, Baltimore, MD; Department of Mental Health, Johns Hopkins University Bloomberg School of Public Health, Baltimore, MD

**Keywords:** COVID-19, crisis leadership, disaster, psychological casualties, resilience

## Abstract

Coronavirus disease (COVID-19) is a “disaster of uncertainty” with ambiguity about its nature and trajectory. These features amplify its psychological toxicity and increase the number of psychological casualties it inflicts. Uncertainty was fueled by lack of knowledge about the lethality of a disaster, its duration, and ambiguity in messaging from leaders and health care authorities. Human resilience can have a buffering effect on the psychological impact. Experts have advocated “flattening the curve” to slow the spread of the infection. Our strategy for crisis leadership is focused on flattening the rise in psychological casualties by increasing resilience among health care workers. This paper describes an approach employed at Johns Hopkins to promote and enhance crisis leadership. The approach is based on 4 factors: vision for the future, decisiveness, effective communication, and following a moral compass. We make specific actionable recommendations for implementing these factors that are being disseminated to frontline leaders and managers. The COVID-19 pandemic is destined to have a strong psychological impact that extends far beyond the end of quarantine. Following these guidelines has the potential to build resilience and thus reduce the number of psychological casualties and speed the return to normal – or at least the new normal in the post-COVID world.

Coronavirus disease (COVID-19) is a “disaster of uncertainty” – one in which there is ambiguity about its nature and trajectory. These features amplify its psychological toxicity and presumably increase the number of psychological casualties it inflicts. Uncertainty is further fueled by lack of knowledge about the lethality of the disaster and its duration, and ambiguity in messaging from leaders and health care authorities. But human resilience can have a buffering effect on the psychological impact. We assert that leaders have not just a prerogative, but rather an obligation to act in ways that foster resilience in those they lead.

During the COVID-19 pandemic, experts have advocated for the goal of “flattening the curve” to slow the spread of the infection.^1^ Our strategy for crisis leadership at Johns Hopkins Medicine may be thought of as a “resilient leadership” approach, which is focused on slowing the rise in psychological casualties by increasing resilience in those they can influence. Resilience, we believe, is a variable that every leader has the opportunity to shape. Its importance will grow as the impact of the pandemic continues, and the subsequent psychological exhaustion and “disillusionment phase” of the psychological response set in.^2^ Recent papers suggest that guidance should be provided to leaders as to the psychosocial aspects and consequences of the pandemic.^3,4^ They add that leadership crisis messaging should take into consideration the psychosocial dimension. This paper describes a novel approach taken at Johns Hopkins Medicine to enhance crisis leadership through the development and promulgation of specific recommendations for leaders at all organizational levels, especially frontline leaders.

Research suggests that at least 4 factors underlie and predict effective crisis leadership: vision for the future, decisiveness, effective communications, and moral authority (following a moral compass).^5-7^ The importance of leadership in times of crisis is a truism. Effective leadership can enhance resilience and may be associated with a lower incidence of psychological casualties.^8,9^ While the 4 factors are empirically linked to perceptions of effective leadership, what is lacking is more specific guidance on how to operationalize them. To address this gap in knowledge transfer, at Johns Hopkins we have developed 9 recommendations, or guiding principles, for implementing the factors in ways that enhance leadership in times of crisis. The recommendations were informed by best practice recommendations,^10,11^ a review of relevant pandemic literature,^12^ as well as our own empirical analysis.^13^ These have been disseminated to frontline leaders and managers in separate pre-recorded, 5-minute modules and posted on the hospital’s intranet website. The modules can be viewed at https://www.hopkinsmedicine.org/joy-at-jhm/office-of-well-being/covid/frontline-crisis-leadership.html.

## STRUCTURE IS THE ANTIDOTE FOR CHAOS

Routines create a sense of continuity and comfort. What can leaders do to create or stabilize routines? Schedule daily briefings to provide updates with any new information. Implement “huddles” at regular intervals, for example, at the beginning and/or end of each shift or operating period within organizations. Provide daily electronic updates.

## LISTEN BEFORE YOU SPEAK

Stress, fear, and anxiety interfere with one’s ability to follow even the most logical guidance. They cause people to revert to reflexive fear-based actions without consideration of the consequences. Cathartic ventilation helps restore deliberative thinking and increases adherence to direction. How can leaders listen more effectively? Allow constituents/staff to voice their personal and professional concerns. Ask open-ended questions – for example, what do you think is happening? What is the worst part of this? Validate, but redirect respectfully when necessary. Do not let the conversation spiral out of control.

## INFORMATION IS AN ANTIDOTE FOR ANXIETY

Information can empower. Keep in mind that there is no such thing as an information vacuum. If leaders aren’t communicating, others are. Information generally comes in 3 variations: anticipatory guidance (what may happen), explanatory guidance (what happened, and why it happened), and prescriptive guidance (recommendations or mandates for action). How can leaders foster the dissemination of relevant information? Provide anticipatory guidance, explanatory guidance, and prescriptive recommendations whenever possible. When commenting on a specific incident, explain: What happened, including causes and physical and psychological reactions; what is being done to remedy the situation; what is being done to prevent or prepare for similar situations in the future.^13^

## TRANSPARENT, TIMELY, AND TRUTHFUL COMMUNICATION IS ESSENTIAL TO MAINTAIN CREDIBILITY

Aristotle asserted that credibility predicts trust and compliance. Trust begins and ends with truth. Trust takes years to build, moments to destroy, and a lifetime to repair. Trust predicts compliance. What can leaders do? Message frequently, anticipating questions and answering them in advance. Repetition is useful: 3–5 times maximizes retention. Remember primacy and recency effects: People remember what they hear first and what they hear last. If you don’t know, say so, but also say when you can provide the information. Stay current with social media regarding concerns, rumors, as well as shared experiences, positive and negative.

## PEOPLE TRUST ACTIONS NOT WORDS

What can leaders do? Demonstrate and model effective actions. Practice the behaviors you prescribe. Follow through on doing what you say you will do.

## EMPOWERMENT IS AN ANTIDOTE FOR FEELING OUT OF CONTROL

Most people do not want to be taken care of nor spoken down to. Rather, they want to be empowered to take care of themselves. What can leaders or managers do to engender an increased sense of control? Ask for input from those who will ultimately implement policies and guidelines. Provide prescriptive guidance on resilience and stress management, giving examples, but again ask personnel about their most effective strategies and encourage sharing among themselves.

## THE PERCEPTION OF SUPPORT IS THE ANTIDOTE FOR ISOLATION

Interpersonal support is the best predictor of resilience.^13^ What can leaders or managers do? Be visible. “Walk among the troops.” Informally monitor the psychological pulse of those you lead. Leaders should check in with constituents/staff frequently. Inform them about the availability of community/institutional resources using as many community channels as possible, and encourage their use. If possible, get training in basic principles of “psychological first aid.”

## COHESIVE GROUPS DO BETTER WITH STRESS AND CHALLENGES THAN NON-COHESIVE GROUPS

What can leaders or managers do to increase the sense of connectedness? Articulate a sense of shared mission, shared identity, and appreciation for the contributions of all team members. Conduct “town hall” meetings or functional unit “huddles” regularly.

## THE MOMENT OF ABSOLUTE CERTAINTY MAY NEVER ARISE

The most egregious failure in leadership is the failure to lead. What can leaders do? Be decisive. Convey an optimistic vision and plan. Do not delay when an important decision is needed.

Johns Hopkins Medicine has provided leadership focused on fostering resilience in its workforce. Incident command centers have been established in every major clinical and functional department, each reporting daily to a unified command center. Virtual town halls have been held regularly to hear concerns from across the health system. Leaders have structured frequent communications to provide the most up-to-date information on COVID-19, and the institutional response. Strong efforts have been made to provide the tools needed to deliver care safely and effectively, and to communicate what is being done to protect health care workers. Daily needs are being supported, with all resources presented on a new platform coordinated by the Office of Well-being. An integrated continuum of mental, emotional, and spiritual support has been established for health care workers at all levels with guidelines for referring internally. Expressions of gratitude have been an integral part of all communications.

Crisis leadership differs from leadership during more normal times. The COVID-19 pandemic is destined to have a strong psychological impact that extends far beyond the end of quarantine, in part, because of the high level of uncertainty associated with this disaster.^14^ This paper has described a novel approach employed at Johns Hopkins to promote and enhance crisis leadership. Following these guidelines has the potential to build resilience and thus reduce the number of psychological casualties and speed the return to normal – or at least the new normal in the post-COVID world.
